# Middle East Respiratory Syndrome and Medical Students: Letter from China

**DOI:** 10.3390/ijerph121013289

**Published:** 2015-10-23

**Authors:** Mengxue Liu, Chengsheng Jiang, Connor Donovan, Yufeng Wen, Sun Wenjie

**Affiliations:** 1 School of Public Health, Wannan Medical College, Wuhu 24100, China; E-Mail: lmxsnow11@sina.com; 2 Maryland Institute for Applied Environmental Health, University of Maryland, MD 20740, USA; E-Mail: cjiang89@umd.edu; 3 College of Business, University of Arkansas at Little Rock, AR 72204, USA; E-Mail: cpdonovan@ualr.edu; 4 School of Food Science, Guangdong Pharmaceutical University, Zhongshan 528458, China; 5 School of Public Health and Tropical Medicine, Tulane University, LA 70112, USA

**Keywords:** MERS, China, medical student, awareness, respiratory infection

## Abstract

*Objectives*: The present study aimed to investigate the knowledge of Middle East Respiratory Syndrome (MERS) among Chinese medical students. *Methods*: A structured questionnaire on MERS was conducted among 214 medical students in China. *Results*: The average correction of the single question varied from 36.0% to 89.7%. There is a significant difference on MERS knowledge among different majors of medical students (*p* < 0.05). Management students scored significantly higher than students of other majors (*p* < 0.05). *Conclusion*: Chinese medical students had good knowledge of MERS. The MERS knowledge score varied among students of different majors. Education on disease control should be included in the school curriculum.

## 1. Introduction 

Middle East Respiratory Syndrome (MERS) is a new, emerging infectious disease that was first reported in 2012 and confirmed with human-to-human transmission in 2013 [1]. On 29 May 2015, the National Health and Family Planning Commission (NHFPC) of China confirmed the first MERS case in China, an imported case from South Korean. MERS is caused by a coronavirus from the same family as the one that triggered China’s deadly outbreak of Severe Acute Respiratory Syndrome (SARS) in 2003. Previous cases have shown that medical students, especially the pre-clinical year medical students, are at high risk of newly emerging diseases, including SARS [2]. While doctors and nurses played a crucial role in the prevention, detection, and containment of SARS [3] most documented “super-spreading events” of SARS occurred in hospitals. The underlying causes remain unclear [4], but it has been suggested that lack of systematic training in emerging infectious diseases could be the cause. A similar case was reported with swine flu [5] 

In China, medical education is comprised of basic medical sciences courses in the first four years and an internship in the fifth year. Although courses regarding preventive medicine are provided to all students, there is no specific course that addresses new emergency infectious diseases. This is concerning because contact with newly emerging infectious diseases such as MERS, H7N9, andH1N1 is not rare in their practice. Chinese medical students’ knowledge on emergency infectious diseases is low. For example, only 35.2% of medical students washed their hands before and 72.5% washed their hands after they physically examined patients in the wards in 2003 before SARS [6] A study from Saudi Arabia addressing the awareness of all procedures concerning prevention of and protection from MERSamong dental students suggested that more information needs to be provided by the authorities for the medical staff [7]. After the first case of MERS was found in China, we designed a study to assess the knowledge of a group of Chinese medical students regarding MERS.

## 2. Method

We conducted a knowledge, attitudes, and practices survey that addressed MERS and its diagnosis and prevention among medical students in May 2015. The study selected individuals to represent the 9000 medical students in Wannan medical college. A multi-stage, random cluster sampling design was used to designate study subjects. All students were divided into grades. Grades 3 and 4 were selected. Each of the above randomly selected grades was divided into multiple majors. The majors of nursing, clinical, management, and public health were randomly selected. Then, one to two classes from these majors were randomly selected to represent the population. Finally, 223 students were selected and 214 responded. The response rate was 95.6%. This study was approved by the Institutional Review Board at Wannan Medical College and followed the tenets of the Declaration of Helsinki. A written form of consent was obtained from all participants.

### 2.1. Measures

A structured questionnaire was adapted from the information released by the National Health and Family Commission of China and a previous MERS-related survey [7]. The questionnaire was comprised of 18 items including demographics and knowledge about MERS in three areas: diagnosis, treatment, and preventive measures. Response options for questions on knowledge about MERS included “Yes”, “No”, and “Don’t know”. A knowledge score was calculated by giving +1 for the correct answer (Yes/No), and 0 for “Don’t know”, with the highest possible score being 18. Higher scores indicated a greater level of knowledge.

### 2.2. Statistical Analysis

Means and standard deviations were used to describe the distribution of continuous variables. Percentages were used to describe the distribution of categorical variables. A chi-square test was performed to examine the differences in educational background and MERS-related knowledge, diagnosis, and prevention between different major groups among medical students. SAS 9.3 (SAS Institute, INC, Cary, NC) was used for all statistical analyses.

## 3. Results

Of 214 participants, 11 were excluded because of missing information on demographic questions. Thus, 203 medical students with a mean age of 21 ± 1.4 (mean ± SD) years participated. Of 203 medical students, 79 (38.9%) were male and 124 (61.1%) were female, 51 (25.1%) students were nursing majors, 61 (30.1%) were clinical majors, 36 (17.7%) were management majors, and 55 (27.1%) were public health majors. Table 1 shows the rate of correct answers, varying from 36.0% to 89.7%. Table 2 shows the difference in knowledge of MERS among the different majors of medical students (*p* < 0.05), e.g., management majors scored significantly higher than students of other majors (*p* < 0.05) (data not shown).

**Table 1:** Respondents’ knowledge of MERS (n = 214), China, May 2015 (Col %).

Questions	Correct Answer	Yes	No	Don’t Know
**Facts**				
1. The first case of human infection with MERS occurred in the Kingdom of Saudi Arabia in 2012.	60.7	60.7	16.8	22.4
2. MERS is caused by a coronavirus.	71.5	71.5	14.5	14.0
3. MERS can be fatal.	89.7	89.7	5.2	5.2
**Transmission**				
4. MERS can spread through close contact with infected persons like those caring for each other and/or living together.	83.6	83.6	8.4	7.9
5. One of the main transmissions of MERS is through saliva and nasal drip from the sick MERS patient.	70.6	70.6	13.1	16.4
**Diagnosis and treatment**				
6. Polymerase Chain Reaction (PCR) can be used to diagnose MERS.	47.2	47.2	24.3	28.5
7. Fever, cough and shortness of breath are typical symptoms of MERS.	74.8	74.8	11.2	14
8. Incubation time for MERS is 14 days.	36.0	36.0	33.6	30.4
9. The symptoms of MERS are very similar to that of influenza.	50.0	50.0	35.5	14.5
10. People with chronic disease are in the high-risk population for MERS.	47.2	47.2	28.5	24.3
11. Antibiotic is an effective medication in the treatment of MERS.	47.7	22.9	47.7	22.4
**Prevention**				
12. MERS can be prevented by good hygiene practices.	68.1	68.1	18.8	13.1
13. Vaccination of MERS is available on the market.	71.4	13.1	71.4	15.5
14. MERS patients should be kept in isolation.	86.0	86.0	8.9	5.1
16. Washing hands with soap and water can prevent MERS transmission	62.1	62.1	28	9.8
17. Avoiding contact with ill people without protection can prevent MERS transmission	83.2	83.2	10.3	6.5
18. Avoiding touching eyes, nose and mouth can prevent MERS transmission	74.8	74.8	12.1	13.1

**Table 2:** The rate of correctly answered questions in each category by major (% correct).

	Nursing	Clinical	Management	Public Health
Facts	58.5%	82.5%	82.5%	75.2%
Transmission	66.1%	83.9%	88.2%	73.6%
Diagnosis and treatment	35.9%	57.8%	64.9%	47.9%
Prevention	63.3%	72.4%	81.2%	74.0%
Total	55.7%	73.7%	78.9%	66.1%

## 4. Discussion

Our study showed that medical students had good knowledge of MERS. Nursing students had the lowest scores in comparison to the students from the other majors, while management students had relatively higher scores compared to the students of the other majors, especially in the categories of “diagnosis and treatment” and “prevention”.

Previous medical knowledge or background might have some impact on the understanding of the disease. The media released the updated information on it. Students from the Department of Management, compared to students in other majors, might be inclined to spend more hours watching TV (especially news programs) or browsing websites in order to follow up with hot social/political topics. The results also reflected the insufficient education on disease prevention and control in the medical school curriculum. The lack of related knowledge among the medical students raises a public concern since they should be prepared with knowledge to confront a potential epidemic and/or pandemic. Although MERS was a newly emergent infectious disease, which people were less familiar with, there are some common principles for dealing with it. The medical staff and public health agencies play a critical role in controlling new emergency infectious diseases. Therefore, it is crucial that healthcare professionals, as well as medical students, be provided with training in controlling infectious diseases [8]. Regarding the contents of the training, the National Health and Family Commission of the People’s Republic of China published the second edition of the MERS Prevention and Control Plan on 5 June 2015 [9]. In addition, the World Health Organization (WHO) and the Centers for Disease Control and Prevention (CDC) also have issued recommendations for the prevention and control of MERS infections in healthcare settings [7]. The prevention of MERS includes hand hygiene, wearing personal protective equipment, and patient placement [10]. In terms of general training, courses such as new emergency diseases, field epidemiology, and tropical medicine can be provided [11,12]. In terms of specific outbreaks, a special course to improve the students’ knowledge of MERS can be provided.

Our study had several limitations. Firstly, lacking the information about students’ sources and the accessibility/availability of MERS-related knowledge (e.g., TV/internet/newspaper, *etc.*), as well as the varying levels of exposure (e.g., media/news time/day) to media, could be a limitation that potentially affects the reliability of the results. For example, the difference of knowledge scores between students in different majors could be confounded by students’ individual habits of accessing information from media. Second, we did not have a general population for comparison. A comparison group consisting of randomly sampled non-medical general population might be needed in the study design to better understand the findings. Future studies addressing those points are warranted. 

Our study had a special public health implication. Special medical education programs on general training on disease control, as well as healthcare guidance on new emergency diseases/outbreaks are warranted. A program needs to be proposed to fill the gap of missing information on MERS for the medical students. It could be a workshop in the classroom or an online program with updated information on new emergency infectious diseases including the topics of prevention, diagnosis, treatment, and patient education. 

## 5. Conclusions

Our findings indicated that Chinese medical students had good knowledge of MERS. The MERS knowledge score varied among students of different majors. Education on new emergency disease prevention and control should be addressed among all medical students.
