# Erratum: Li, M., *et al*. Middle East Respiratory Syndrome and Medical Students: Letter from China. *Int. J. Environ. Res. Public Health* 2015, *12*, 13289–13294

**DOI:** 10.3390/ijerph13030335

**Published:** 2016-03-18

**Authors:** International Journal of Environmental Research and Public Health Editorial Office

**Affiliations:** MDPI, Klybeckstrasse 64, Basel CH-4057, Switzerland; ijerph@mdpi.com; Tel.: +41-61-683-77-34

We wish to make the following correction to the published paper [1]. The corresponding author’s name is incorrect. It should be “Wenjie Sun”, not “Sun Wenjie”.

We apologize to the authors of the article and readers of the journal for any inconvenience.
